# Unleashing the immune modulatory potential of *Leishmania amazonensis-*derived extracellular vesicles in American cutaneous leishmaniasis

**DOI:** 10.3389/fmolb.2025.1593363

**Published:** 2025-09-22

**Authors:** Bruna Eugênia de Freitas, Armanda Rodrigues, Joana Palma-Marques, Juliana Inês Weber, Ana Valério-Bolas, Rodrigo Pedro Soares, Ana Claudia Torrecilhas, Micheli Ferla, Munira Muhammad Abdel Baqui, Raul Alexander Gonzales Cordova, Graça Alexandre-Pires, Isabel Pereira da Fonseca, Hélida Monteiro de Andrade, Gabriela Santos-Gomes

**Affiliations:** ^1^ Global Health and Tropical Medicine, GHTM, LA-REAL, Instituto de Higiene e Medicina Tropical, IHMT, Universidade Nova de Lisboa, Lisboa, Portugal; ^2^ Instituto de Ciências Biológicas, ICB, Universidade Federal de Minas Gerais, Fundação Oswaldo Cruz, Belo Horizonte, Brazil; ^3^ Biotechnology Applied to Pathogens Group (BAP), Instituto René Rachou/FIOCRUZ, Belo Horizonte, Brazil; ^4^ Químicas e Farmacêuticas, Universidade Federal de São Paulo (UNIFESP), Instituto de Ciências Ambientais, Belo Horizonte, Brazil; ^5^ Department of Cellular and Molecular Biology and Pathogenic Bioagents, Ribeirão Preto Medical School, University of São Paulo, USP, Ribeirão Preto, Brazil; ^6^ CIISA - Centre for Interdisciplinary Research in Animal Health, Faculty of Veterinary Medicine, University of Lisbon, Lisbon, Portugal; ^7^ Associate Laboratory for Animal and Veterinary Sciences (AL4AnimalS), Vila Real, Portugal

**Keywords:** American cutaneous leishmaniasis, neglected skin disease, leishmaniaamazonensis, extracellular vesicles, immune modulation, skin homeostasis

## Abstract

**Introduction:**

American cutaneous leishmaniasis (ACL) constitutes a neglected skin disease that causes severe disability and significant social stigma for millions of people each year. This parasitic infection is caused by several species of the protozoan *Leishmania*, including *Leishmania amazonensis*. There is therefore an urgent need to develop effective new tools to control ACL, primarily due to the limitations of current prophylactic and therapeutic strategies, which are exacerbated by the growing burden of the disease and its social impact. In recent years, scientific research has focused on extracellular vesicles (EVs), which are lipid-enclosed rounded nanostructures that carry macromolecules to recipient cells and are part of eukaryotic biology. The role of *Leishmania*-derived EVs in host pathogenesis has attracted considerable attention among researchers, with studies suggesting that EVs may play a key role in modulating the host immune response. Therefore, this study examined the immunogenicity and protein cargo of EVs shed by *L. amazonensis,* exploring their effect on immune activation in the murine macrophages (MΦs) lineage.

**Methods:**

Nanoparticle tracking analysis, microscopy, proteomic methodologies, colorimetric assays, serological immune methods, PCR, and multiparametric flow cytometry were employed.

**Results:**

EVs derived from *L. amazonensis* cultured promastigotes contain key components, such as the 63 kDa surface glycoprotein, intracellular heat shock protein 70, and α-type proteasome subunit, which may be involved in parasite survival. Moreover, EVs are recognized by mouse- and human-specific antibodies, indicating that they have the potential to elicit humoral immune responses and can be inactivated by host-specific antibodies. Depending on the concentration, EVs can drive MΦs to express MHC molecules that are essential for antigen presentation to T lymphocytes, thereby being able to promote a cellular immune response. EVs favor IL-1β^+^MΦs contraction, and low nitric oxide production, and activate the arginase pathway to produce urea along with the generation of proinflammatory cytokines. This MΦs modulation may support parasite control through the specific activation of T cells while preserving skin homeostasis, thereby reducing the pathology associated with *L. amazonensis* infection, which causes ACL and leads to the development of chronic disease.

**Discussion:**

Thus, this study’s findings suggest that although *L. amazonensis*-derived EVs can trigger MΦs activation, favoring a pro-inflammatory immune response, they also have the potential to ensure parasite survival while limiting host pathogenesis. This can be advantageous for parasite transmission and essential for completing the parasite life cycle.

## 1 Introduction

Leishmaniasis is a group of diseases caused by protozoa belonging to the *Leishmania* genus. These protozoa are transmitted to humans and other mammals through the bite of infected female sandflies during their blood meal. There are at least three main clinical manifestations of these diseases: visceral leishmaniasis (VL), cutaneous leishmaniasis (CL), and mucocutaneous leishmaniasis. Human VL is mainly caused by *Leishmania donovani* in the Indian subcontinent and East Africa, and by *Leishmania infantum* in the Mediterranean region, the Middle East, China, parts of Asia and East Africa, and Central and South America. Other species may occasionally cause visceral clinical features, as is the case of *Leishmania amazonensis* (de Souza et al., 2018; Porto et al., 2022). CL is caused by several species belonging to the *Leishmania* genus, specifically the subgenus *Leishmania* or *Viannia*. I,n the Old World the main species responsible for CL occurrence are *L. major*, *L. aethiopica,* and *L. tropica*, while in the Americas are *L. mexicana*, *L. amazonensis*, *L. braziliensis*, *L. venezuelensis*, *L. shawi*, *L. guyanensis*, *L. panamensis,* and *L. peruviana* (Gabriel et al., 2019).

Leishmaniasis is classified as a neglected disease and is responsible for the death and disability of millions of individuals per year, evidencing a need for increased attention. This parasitic disease is considered endemic in 99 countries, 90 of which are endemic for CL, 80 for VL, and 71 for both clinical forms. In 2022, about 206,000 new cases of CL were recorded worldwide, the majority of which (85%) were registered in Afghanistan, Algeria, Colombia, Brazil, the Islamic Republic of Iran, Iraq, Peru, and the Syrian Arab Republic (Ruiz-Postigo et al., 2023). However, due to probable underreporting, it is estimated that 600,000 to 1,000,000 new cases of CL and about 100,000 cases of VL occur annually worldwide (Mann et al., 2021; Leishmaniasis, 2023).

Macrophages (MΦs) are phagocytic cells and the final host cells of the *Leishmania* parasites (Pereira et al., 2019). Within MΦs phagolysosomes, *Leishmania* promastigote differentiates into the amastigote form, which is a small, rounded, and immobile form that replicates and can resist intracellular defense mechanisms. Parasite ligands that bind to surface, endosomal, or cytoplasmic innate immune receptors can activate downstream immune pathways, triggering cytokine generation.

Moreover, the intracellular amastigote shapes MΦs immune activity by driving their differentiation into different functional phenotypes (Gabriel et al., 2019; Santos-Mateus et al., 2016). MΦs with pro-inflammatory and microbicide functions can produce pro-inflammatory cytokines such as interleukin (IL)-1β, IL-12, and TNF-α. and exhibit cytotoxicity activity driven by the expression of nitric oxide-synthetase 2 (NOS2), which culminates in the production of nitric oxide (NO). These MΦs are effective at eliminating pathogens, but detrimental to the wound-healing process. In contrast, MΦs exhibiting pre-resolving inflammation and tissue repair functions produce anti-inflammatory cytokines, such as IL-10, and express arginase, leading to urea production towards the activation of the arginine metabolic pathway. These MΦs promote cell proliferation and collagen production (Rodríguez-Morales and Franklin, 2023), aiding healing and tissue repair. MΦs also play a role as non-professional and professional antigen-presenting cells (APCs) by processing and presenting pathogen-derived antigens complexed with class I and class II molecules of major histocompatibility complex (MHC) to T cells, thereby modulating cellular immune response.

Extracellular vesicles (EVs) are a generic term used for nanosized lipid bilayer particles naturally released by cells, which lack a functional nucleus and are unable to replicate (Welsh et al., 2024). In recent years, the role of EVs shed by *Leishmania* parasites has been studied, and research findings point to an immunomodulatory function favorable to infection. In 2020, Nogueira and collaborators (Nogueira et al., 2020) demonstrated that *L. amazonensis*-derived EVs induced murine MΦs to produce NO, TNF-α, IL-6, and IL-10, via the activation of toll-like receptor (TLR) 4 and TLR2 downstream pathways. Moreover, EVs-based vaccines have been studied for various diseases, such as cancer, and viral, bacterial, and parasitic infectious diseases (Montaner-Tarbes et al., 2021; Chowdhury et al., 2024; Peregrino et al., 2024; Zhang et al., 2024). In a previous publication involving an experimental immunization model of CL, *L. amazonensis* EVs were identified as potential components for active immunization, conferring partial protection and inducing polarization of the host’s immune response towards a protective type T helper (Th1) immune response (Dupin et al., 2021). Furthermore, proteomic studies have shown that the amount, morphology, distribution, and protein load of *L. infantum* EVs differ between drug-resistant and sensitive strains (Douanne et al., 2020). These and other studies point out the importance of researching the interaction between *Leishmania-*derived EVs and the host immune response, indicating that EVs and their proteins could be promising candidates for vaccine development, as well as targets for innovative therapeutics.

Examining the proteome and immunoreactivity of *Leishmania* EVs can provide new insights into the significance of these vesicles to parasite survival throughout its life cycle. Furthermore, this study can shed light on the impact of EVs on MΦs' activity. Therefore, this work analyzes the protein signature and immunogenicity of EVs purified from *L. amazonensis* cultured promastigotes, as well as their effect on immune activation and functionality of murine MΦs.

## 2 Materials and methods

### 2.1 *Leishmania amazonensis* parasites and preparation of soluble antigen

The *L. amazonensis* strains MHOM/BR/1973/M2269, MHOM/BR/1987/BA125, and MHOM/BR/1989/BA336 (Silva et al., 2021) were used to purify EVs and produce soluble antigens. The EVs were then used for protein analysis, to assess immunoreactivity, and to examine their effect on MΦs' activity. The parasites were grown in axenic conditions in M199 medium (Medium 199, Earle’s Salts, Gibco™), supplemented with 10% of heat-inactivated fetal bovine serum (hiFBS) (56 °C for 30 min) and 1% penicillin/streptomycin (PenStrep, 10,000 IU penicillin.mL^-1^ and 10 mg streptomycin.mL^-1^, Sigma-Aldrich), at 24 °C. To prepare soluble antigen, *L. amazonensis* cultured promastigotes in the stationary growth phase were centrifuged at 1800 *g* for 15 min at 4 °C. The resulting pellet was then resuspended in sterile 1× phosphate-buffered saline (PBS) and washed twice. After the last wash, the pellet was resuspended in distilled water. Then, parasites were subject to six cycles of freezing (−20 °C) and thawing at room temperature, followed by centrifugation to remove the insoluble material. Antigen concentration was estimated using a spectrophotometer (NanoDrop 1000, Thermo Fisher Scientific, United States).

### 2.2 *Leishmania amazonensis* hyperimmune serum

Six-week-old BALB/c mice of both genders were used to obtain *L. amazonensis* hyperimmune serum. The mice were acquired from the Central Bioterium of the Federal University of Minas Gerais (UFMG) and housed in the animal facility of the Department of Parasitology at the Biological Science Institute (ICB). The animals were kept under appropriate environmental and nutritional conditions following the requirements of the National Council for The Control of Animal Experimentation (CONCEA), as regulated by The Arouca Law 11,794 of the Brazilian Federal Constitution. All experiments involving mice and the associated study protocols were approved by the Institutional Animal Care and Use Committee (IACUC) of the Federal University of Minas Gerais (protocol number 269/2018). The mice were separated into two groups. Six mice were used as controls, and seven mice were inoculated by the intraperitoneal route with 1 × 10^6^ *L. amazonensis* promastigotes (PH8 strain), which were harvested from the stationary phase of growth in M199 medium supplemented with hiFBS. After 8 weeks of infection, mice were subjected to general anesthesia using ketamine and xylazine and then bled from the brachial plexus. Whole blood samples were obtained for the subsequent collection of sera. The spleen was removed to make cytological imprints that were stained with Giemsa dye and examined under an optical microscope to confirm infection by observing *L. amazonensis* amastigote forms. Whole blood samples collected from both groups of mice were centrifuged at 2,000 × *g* for 15 min at room temperature. The serum was separated into 30–50 μL aliquots and stored at −80 °C for later use. The level of anti-*Leishmania* antibodies was evaluated by ELISA, using plates coated with *L. amazonensis* soluble antigen and serum titrations of 1:25, 1:50, 1:100, and 1:200. The cut-off used was determined as the mean absorbance of the uninfected group (negative control), excluding the white absorbance, plus three times the standard deviation.

### 2.3 Purification of EVs released by *Leishmania amazonensis*

Two methods were employed to purify EVs from axenic cultures of *L. amazonensis* promastigotes: ultracentrifugation, which enables the isolation of pure EVs from a large sample volume, and a polymer-based precipitation approach using the Exosome Isolation Reagent (Invitrogen), as described by Weber *et al.* (Weber et al., 2023), which allows a higher yield of EVs to be recovery.

A suspension of 2 × 10^7^ parasites.mL^-1^ was incubated for 72 h at 26 °C in M199 axenic culture medium supplemented with 10% exosome-depleted bovine fetal serum (FBS ExoFree, Thermo Fischer Scientific). The supernatant was recovered by centrifugation at 1,000 × *g* for 15 min. Total Exosome Isolation Reagent was then added to the supernatant of *L. amazonensis* cultures and incubated overnight at 4 °C. The suspension was then centrifuged at 10,000 × *g* for 60 min at 4 °C, and the EVs retained in the pellet were resuspended in sterile PBS. EVs’ total protein concentration was estimated using a Qubit® 4 Fluorometer (Thermo Fisher Scientific). The isolated EVs were stored at −80 °C and used for immune studies.

To harvest EVs by ultracentrifugation (Fernandez-Becerra et al., 2023), the culture supernatant was filtered using 0.22 μm sterile filters and then centrifuged four times at 4 °C in an ultracentrifuge (Sorvall® Ultra Pro 80) as follows: (i) 500 × *g* for 10 min; (ii) 1,500 × *g* for 10 min; (iii) 100,000 × *g* for 10 min, and (iv) 1000,00 × *g* for 2 hours. The resulting pellet was resuspended in serum-free M199 medium, and the total protein concentration was estimated by fluorometry. These EVs were stored at −80 °C and used for morphological and protein-based assays.

### 2.4 Morphology, size, and concentration of *Leishmania amazonensis*-derived EVs

The morphology of EVs shed by *L. amazonensis* promastigotes was examined using transmission electron microscopy (TEM). EVs were fixed in Protein fractions were separated by sodium dodecyl sulfate-polyacrylamide gel electrophoresis (SDS-PAGE) and analyzed using immunoblotting and mass spectrometry Karnowsky’s fixative solution for 30 min at 4 °C, after which they were washed three times with 0.1 M cacodylate buffer. The fixed EVs were then deposited on carbon-coated 300-mesh copper grids and left to settle for 20 min. The grids were then negatively stained with 5% (v/v) uranyl acetate for 5 min. The EVs were observed under a Jeol JSM-6610 LV microscope at the Multiuser Electron Microscopy Laboratory (LMME, Ribeirão Preto Medical School, USP), and digital images were captured.

The size and concentration of the purified EVs were analyzed using nanoparticle tracking analysis (NTA, NanoSight NS3000 equipment, Malvern Instruments Ltd., Malvern, UK, equipped with a 405 nm CCD laser camera, and NTA 2.3 software). Each sample of EVs’ diluted 1:100 in PBS was recorded in triplicate for 30 s.

### 2.5 Proteins of *Leishmania amazonensis*-derived EVs

To reveal the protein composition of EVs shed by *L. amazonensis* promastigotes protein fractions were separated by sodium dodecyl sulfate-polyacrylamide gel electrophoresis (SDS-PAGE) and analyzed using immunoblotting and mass spectrometry. For this, 20 μg of pre-heated EVs (95 °C in a thermoblock for 5 minutes) was diluted in 2× sample buffer (100 mM Tris/HCl pH 6.8, 4% SDS, 0.2% bromophenol blue, 20% glycerol) and loaded onto a 12% 1D SDS-PAGE gel. Prestained broad range standards (BIO-RAD) were also loaded onto the gel as molecular weight markers. The separated protein fractions were further processed for analysis. The fractions were stained with Comassie Blue G250 (Sigma-Aldrich) and then transferred onto nitrocellulose membranes or directly used for mass spectrometry to identify the proteins carried by EVs. The molecular masses of the bands on the acrylamide gel were estimated by comparing them with the molecular weight markers using the GelAnalyzer 23.1 software.

The presence of 70 kDa heat shock protein (HSP70) in EVs was investigated using Western blot. An in-house recombinant HSP70 *Leishmania* protein (10 μg) was used as a positive control. Briefly, the synthetic *Leishmania* HSP70 gene was inserted into the pET28a-TEV vector. The pET28a-TEV construct was then transformed into the bacterium *Escherichia coli* BL21 (DE3) for protein expression. To induce expression, kanamycin (50 μg.mL^-1^) was added to the culture medium along with the plasmid pre-inoculum (one colony in 25 mL of medium). The culture was stirred at 37 °C until an optical density of 0.4 was reached at 600 nm. Protein expression was then induced by adding 1 M isopropyl-thiogalactoside, after which the culture was incubated for 4 h. The bacteria were then lysed using sonication (4 cycles of 20 s at 40 W, alternating with 20 s on ice) to release the protein content. The recombinant HSP70 was then purified using high-pressure chromatography with nickel affinity columns (ion exchange), ensuring a highly purified protein for further use. During this process, only molecules with a histidine tail that were added to the recombinant protein through the pET28a-TEV vector were selectively bound and eluted from the column. The purified antigen underwent dialysis on a membrane to remove the urea, followed by lyophilization.

EVs’ protein fractions were transferred to a nitrocellulose membrane using a Semi-Dry Blotter (TE 77 PWR, Amersham Biosciences) at a voltage of 1.6 mA/cm^2^ for 45 min. The membrane was then blocked for 1 hour with 5% milk powder, rinsed rapidly with distilled water, and incubated at 4 °C for 8 h with anti-HSP70 Rabbit Monoclonal Antibody (diluted 1:1500, Sigma-Aldrich) diluted in blocking solution. After three washes, the membrane was treated with a secondary anti-rabbit antibody conjugated directly with horseradish peroxidase (Goat Anti-rabbit IgG antibody, Sigma-Aldrich), diluted at 1:300, for 90 min at room temperature. The chemiluminescence reaction was performed using Thermo Fisher’s ECL (enhanced chemiluminescence) kit, and digital images were captured with the ImageQuantTM LAS500 digital CCD camera system. The molecular mass of the protein bands was estimated following the manufacturer’s instructions (Bio-Rad).

A simplified proteomic approach was employed to identify additional proteins carried by EVs. Thus, EVs proteins were separated using 12% SDS-PAGE, stained with Coomassie G250, and the resulting bands were manually excised and transferred to microtubes. The excised gel fragments were destained with three sequential washes in a solution of 25 mM ammonium bicarbonate and 50% acetonitrile at pH 8 before being dehydrated with acetonitrile and dried in the Eppendorf® Concentrator Plus centrifuge (Sigma-Aldrich). Proteolytic digestion was performed by adding 10 μL of 20 ng.μL^-1^ trypsin (Promega) in 25 mM ammonium bicarbonate, after which the mixture was incubated for 16 h at 37 °C. The resulting peptide supernatant was collected in siliconized tubes. To maximize recovery, the gel fragments were washed twice with a solution of 50% acetonitrile and 5% trifluoroacetic acid for 30 min. The resulting supernatant was added to the siliconized tubes, and the final volume was reduced using an Eppendorf® Concentrator Plus centrifuge. These concentrated peptide solutions were desalted using reverse-phase C18 columns (Zip-Tip®, Millipore), and the peptides were eluted from these columns using a solution of 50% acetonitrile and 0.1% trifluoroacetic acid. Mass spectrometry (MS) and MS/MS data were acquired for EVs using a MALDI-TOF/TOF Ultraflex III spectrometer (Bruker Daltonics). The 3D ion trap mode was employed with scan ranges of 300 up to 800 m/z for MS and 100 up to 3,000 m/z for MS/MS data. Collision-induced dissociation was applied at a resolution range of 50 ppm–150 ppm for fragmentation patterns. Standard mass spectrometric conditions included a dry gas flow of 3 L.min^-1^ at a source temperature of 200 °C and a capillary voltage (HV) of 1500 V. Samples were dissolved in 70 μL of water containing 0.1% formic acid, and 4 μL of this solution was introduced into the mass spectrometer.

Data acquisition was analyzed using Hystar, version 3.2 (Bruker Daltonics, Germany), and data processing was performed employing Data Analysis version 4.0 (Bruker Daltonics) software. The processed data were then converted to the mzXML format using CompassXport, version 3.0 with 64-bit precision (Bruker Daltonics). Protein was identified through PEAKS software (version 7.0 Bioinformatics Solutions Inc., Canada), which matched sequences against the UniProt_SwissProt_06-02-2022 database (567.483 entries) and UniProt_Leishmania_2022 database (142.262 entries).

The presence of the glycoprotein of 63 kDa (gp63) in *L. amazonensis*-derived EVs was evaluated using a dot-blotting. The nitrocellulose membrane was sensitized directly with EVs (5 μg) and left to dry at 4 °C for 8 h. The membrane was then immersed in a blocking solution (5% milk powder in 1× PBS) for 2 h at room temperature. After a brief rinse with distilled water, the membrane was incubated with *Leishmania* anti-gp63 primary antibody (Anti-Leishmania Major Surface Protease gp63 Monoclonal Antibody, Cedarlane) at a dilution of 1:500 in blocking solution for 1 hour at room temperature. After three washes with washing solution (0.5% Tween 20 in 1× PBS) under gentle agitation for 5 minutes each, the membrane was incubated for 1 hour at room temperature with a secondary anti-mouse antibody (rabbit anti-mouse IgG whole molecule, Sigma-Aldrich) directly conjugated with horseradish peroxidase and diluted in blocking solution at 1:500. The membranes were then washed three times, and the luminol-based chemiluminescent substrate (Pierce™ ECL Western blotting Substrate, Thermo Fisher Scientific) was added. In parallel, parasite antigens and EVs treated with dithiothreitol digest buffer were also analyzed. The resulting images were captured using the CCD digital camera system (ImageQuant™ LAS 500 device, GE Healthcare Bio-Sciences AB).

### 2.6 Immunogenicity of *Leishmania amazonensis*-derived EVs

The immunogenicity of EVs was investigated using Western blot. The study involved (i) a pool of ten serum samples from patients presenting clinical signs compatible with ACL that were positive for indirect immunofluorescence reaction, (ii) a pool of ten sera from healthy individuals that were serologically negative by rK39 ELISA, (iii) hyperimmune serum obtained from seven mice experimentally infected with *L. amazonensis,* and (iv) sera from six healthy uninfected mice. The human serum samples were provided by the Laboratório de Pesquisa Clínica at the Rene Rachou Research Center (FIOCRUZ, Belo Horizonte, Brazil). Ethical clearance for the study was obtained from the FIOCRUZ Institutional Review Board, the National Research Ethics Committee, and the Brazilian Ethics Platform CAAE: 30429114.1.3001.5091.

SDS-PAGE was employed to separate the protein fractions of EVs, which were then transferred to a nitrocellulose membrane and blocked with 5% milk powder for an hour. The positive control was *L. amazonensis* soluble antigen (20 μg), while the negative control was M199 medium supplemented with 10% hiFBS. The membrane strips were then incubated for 8 h at 4 °C with mouse hyperimmune sera (1:100), sera from uninfected mice (negative control), ACL patient sera (1:100), and sera from healthy humans (negative control). Following incubation, the strips were washed and incubated for 1 hour with a secondary antibody conjugated to horseradish peroxidase (1:500, Sigma-Aldrich). After washing, substrate was applied to the strips, and digital images were acquired using a CCD camera system. The molecular mass of the immunoreactive protein fractions was estimated by establishing a standard calibration curve using a range of prestained protein standards (Bio-Rad), according to the manufacturer’s instructions.

### 2.7 Effect of *Leishmania amazonensis* EVs on MΦs' immune modulation

To analyze the influence of *L. amazonensis* EVs on the activation of murine MΦs, the macrophage-like mouse cell line (P388D1) was cultured in suspension in Roswell Park Memorial Institute (RPMI) 1640 medium supplemented with 10% heat-inactivated exo-free FBS and 1% PenStrep at 37 °C in a humidified atmosphere with 5% CO_2_. Fresh medium was added every two or 3 days, maintaining an average cell density of 1 × 10^6^ MΦs.mL^-1^. A total of 2 × 10^5^ cells per well were then stimulated with EVs derived from the MHOM/BR/1973/M2269 strain at two different concentrations: 7.09 μg.μL^-1^ and 3.54 μg.μL^-1^. In parallel, MФs were exposed to stationary phase *L. amazonensis* promastigotes (at a ratio of 3 parasites per MΦ) or stimulated with soluble *L. amazonensis* antigen (40 μg.mL^-1^). Resting MФs were used as a negative control, and MΦs stimulated with phorbol-12-myristate-13-acetate (PMA, 30 μM) were used as a positive inflammatory control. The plates were incubated for 24 h at 37 °C in a humid atmosphere containing 5% CO_2_. After incubation, the supernatants were collected for urea quantification by colorimetric assay, and the cells were obtained for confirmation of metabolic activity by resazurin reduction assay, immunophenotyping using multiparametric flow cytometry, and transcriptional studies of innate receptors and cytokines.

### 2.8 MΦs viability assessed through metabolic activity

A resazurin reduction assay was performed on MΦs stimulated with EVs for 24 h to assess cell viability, evaluating their metabolic activity. Unstimulated MΦs were used as the positive control, and MΦs incubated with 2% paraformaldehyde as the negative control. The metabolic activity of MΦs stimulated with soluble *L. amazonensis* antigens, or PMA, and MΦs exposed to *L. amazonensis* promastigotes was also analyzed.

Resazurin sodium salt was dissolved in ammonium hydroxide at 3.9 mM. The resazurin working solution was then diluted in 1× PBS to a final concentration of 0.033 μg.mL^-1^, and 25 μL of this solution was added to each well containing MΦs. The plates were then incubated for 1 hour at 37 °C in a humid atmosphere containing 5% CO_2_. Fluorescence intensity was measured at 595 nm following excitation at 535 nm using the Concert™ fluorimeter (TRIAD™ version 2.1.0.17), and the data were analyzed employing the Concert™ Software (TRIAD™ version 2.1.0.17). Macrophage numbers were accessed based on fluorescence intensity.

### 2.9 Gene expression of innate receptors and cytokines in MΦs

Gene expression of innate immune receptors and cytokines was analyzed in MΦs stimulated with *L. amazonensis*-derived EVs to assess the influence of EVs on MΦ immune activity compared with parasitic soluble antigen or exposure to *L. amazonensis* parasites. Specifically, the gene expression of Toll-like (TLR2 and TLR9) and NOD-like (NOD1 and NOD2) innate receptors was analyzed along with key cytokines involved in the immune response. These included the anti-inflammatory *il10* and pro-inflammatory cytokines [*il1b, tumor necrosis factor (tnf) α,* and the *il12* heterodimer *p40*]. Total RNA was extracted from MΦs, and cDNA synthesis was performed as described previously (Weber et al., 2023). Mouse-specific primers with efficiencies ranging from 90% to 100% (Supplementary Table S1) were used for semiquantitative real-time PCR (qPCR). DNA amplification was conducted using NZYSpeedy qPCR Green Master Mix (2×) (NZYtech) under the following conditions: an initial denaturation step at 95 °C for 1 min, followed by 39 cycles of denaturation at 94 °C for 2 s, annealing for 1 s, and DNA extension at 50 °C for 5 s. The housekeeping gene hypoxanthine phosphoribosyl transferase (HPRT) was used to establish the baseline gene expression level (ΔCt). Resting cells (non-stimulated MΦs) were employed as the negative control for relative quantification (∆∆Ct), and the final relative mRNA expression levels were calculated using the 2^−ΔΔCT^ method (Pfaffl, 2001).

### 2.10 MΦ immunophenotyping

The MΦs collected from the microplates were washed with 500 μL of 1× PBS at 300 × *g* for 10 min at room temperature. The cells were then fixed with 2% paraformaldehyde for 20 min at 4 °C in the absence of light, after which were centrifuged at 300 *g* for 10 min at 4 °C. Anti-MHCI and anti-MHCII monoclonal antibodies directly conjugated with fluorochromes were added to cells and left to incubate in the dark for 30 min at 4 °C. The cells were then washed with 200 μL of 1× PBS containing 2% albumin, incubated with 100 μL of permeabilization buffer (1× PBS 2% albumin and 0.5% Tween 20), and labeled with anti-IL-1β monoclonal antibody directly conjugated with a fluorochrome (Supplementary Table S2). This permeabilization step facilitates the passage of antibodies into the intracellular space, enabling the labeling of intracellular molecules. Unbound antibodies were removed by washing MΦs with 1× PBS (300 × *g* for 10 min at 4 °C). The cells were then resuspended in 200 μL of 1× PBS 2% albumin and acquired by the flow cytometer (CytoFLEX cytometer, Beckman Coulter Life Sciences). For calibration, samples labeled with a single fluorochrome and unlabeled samples were used to adjust voltage and fluorescent settings. During cell acquisition, a maximum of 10,000 events were recorded, and an FSC-H vs*.* SSC-H gate was applied to exclude debris and pyknotic cells, as well as the large debris (off-scale). A singlet gate based on pulse geometry (FSC-H vs*.* FSC-A) was then applied to exclude doublets and ensure non-clumping analysis. The data were processed, and the frequency of IL-1β^+^MΦs and the mean fluorescence intensity (MFI) of MHCI and MHCII molecules were analyzed by Flowjo V10 software (Tree Star Inc., United States).

### 2.11 Urea and nitric oxide production by murine MΦs

To evaluate the final products resulting from the activation of L-arginine pathway, MΦs supernatants were centrifuged to remove cell debris and used to quantify urea and NO production through the Urea Assay Kit (BioChain®, United States) and Nitrate/Nitrite colorimetric assay kit (Cayman Chemical, United States), respectively, according to the manufacturer’s instructions. The chromogenic reagent present in the assay reacts specifically with urea, developing a colorimetric complex that was analyzed by spectrophotometry at a wavelength of 430 nm (TRIADTM 1065 fluorimeter, DYNEX Technologies). The color intensity is directly proportional to the urea concentration in the sample. The nitrate/nitrite concentration was determined using a two-step colorimetric process that reduces nitrate to nitrite using the nitrate reductase, followed by the conversion of the total nitrite into the purple azo compound. As nitrite and nitrate are stable end-products of NO metabolism, their levels were assessed as indirect indicators of NO production. Absorbance was measured at 540 nm, and the values were normalized to RPMI supplemented medium.

### 2.12 Data analysis

The results of at least three independent experiments, each evaluated in duplicate, were analyzed using GraphPad Prism 10 (GraphPad Software Inc.). Data normality was assessed using the Kolmogorov-Smirnov test. As the data did not follow a normal distribution, the two-tailed Wilcoxon matched-pairs signed-rank test was employed for pairwise comparisons. A significance level of 5% (*p* < 0.05) was used to indicate statistical significance.

## 3 Results

Due to their similarity and higher infectivity (Silva et al., 2021), the MHOM/BR/1987/BA125 and MHOM/BR/1989/BA336 strains were mainly employed in the characterization of EVs. EVs derived from the MHOM/BR/1973/M226 strain were primarily used in immunological assays, supporting the hypothesis that these EVs may shape MΦs' immune response without triggering a strong inflammatory effect. This is particularly relevant in the context of *L. amazonensis* infection, where excessive inflammation can exacerbate cutaneous lesions.

### 3.1 *Leishmania amazonensis* axenic promastigotes shed EVs

**FIGURE 1:**
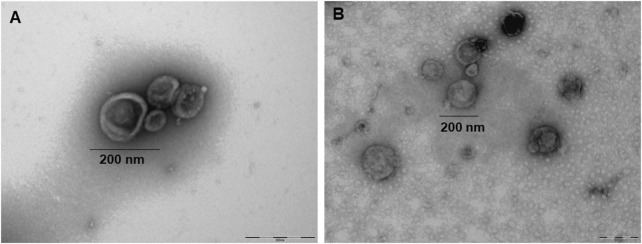
EVs-derived from *Leishmania amazonensis* promastigotes. Representative TEM images of EVs purified from axenic cultures of MHOM/BR/1989/BA336 **(A)** and MHOM/BR/1987/BA125 **(B)** strains. A corona-like layer (arrow) can be observed surrounding isolated EVs **(A)** Scale bar: 200 nm.

EVs shed by cultured promastigotes of *L. amazonensis* strains (MHOM/BR/1987/BA125 and MHOM/BR/1989/BA336) (Figures 1A,B) are rounded in shape and appear to be coated with a corona-like layer (Figure 1A). This sharply defined peripheral margin appears to be less electron-dense than the vesicle and is consistent with the previously described corona-like protein layer in EVs derived from mammalian cells (Wolf et al., 2022). The size of purified EVs ranges from 40 to 378 nm, with an average ranging between 198 nm (198.3 ± 1.9 nm for MHOM/BR/1989/BA336) and 226 nm (226.4 ± 5.4 nm for MHOM/BR/1987/BA125).

**FIGURE 2:**
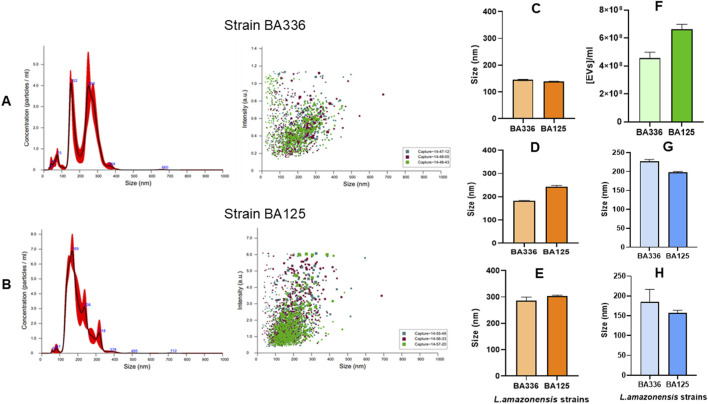
Size, concentration, and intensity of EVs released by *Leishmania amazonensis* strains. Size vs*.* concentration or intensity of EVs purified from *Leishmania amazonensis* strains MHOM/BR/1989/BA336 **(A)** and MHOM/BR/1987/BA125 **(B)** are shown as determined by NTA and represented by histograms (mean ± SD) and dot plots. Three particle captures of 60 s each are registered in dot plots using different colors to visualize replicate variability. The size of EVs in 10% **(C)** 50% **(D)** and 90% **(E)** percentiles, concentration of EVs per ml **(F)** and the mean **(G)** and mode **(H)** of EVs are represented by bar graphs.

The parasites (MHOM/BR/1989/BA336) released a large population of EVs with sizes ranging from 152 to 250 nm (Figure 2A), which are compatible in size with microvesicles. The concentration of these peaks varied between 3.7 and 4 × 10^8^ EVs.mL^-1^. Two additional peaks were identified by NTA, corresponding to EVs ranging in size from 42 nm to 100 nm (average size of 75 nm), which are highly compatible with tiny and large exosomes. In contrast, MHOM/BR/1987/BA125 strain showed a predominant peak of EVs with a size of 169 nm and a concentration of 7.5 × 10^8^ EVs.mL^-1^ (Figure 2B).

Regarding size distribution, 10% of the total volume contains EVs smaller than 145 nm (Figure 2C). Half of the EVs’ population was inferior to 242 nm, and 90% of the total volume contained EVs smaller than 303 nm (Figures 2D,E). These data confirm that *L. amazonensis*-derived EVs include both exosomes and microvesicles. The average density of EVs ranged from 4.57 × 10^8^ to 6.64 × 10^8^ particles.ml^-1^ (Figure 2F) and the mean size varied between 198.3 and 226.4 nm (Figure 2G). The most abundant EVs present 157.2 and 185.2 nm (Figure 2H).

Although differences were found in the size, concentration, and density of EVs shed by the 2 *L. amazonensis* similar strains, concerning virulence and infectivity, these results indicate that cultured promastigotes of both strains mainly emit large EVs, but also shed smaller EVs, less than 100 nm.

### 3.2 Cargo of *Leishmania amazonensis*-derived EVs includes HSP70, gp63, and the α-type proteasome subunit

**FIGURE 3:**
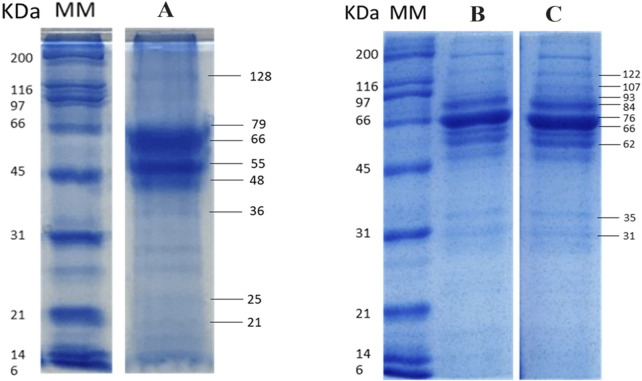
Protein fractions profiles of *Leishmania amazonensis-*derived EVs. Digital images of electrophoresis in gel acrylamide (SDS-PAGE) of EVs purified from *Leishmania amazonensis* strains MHOM/BR/1973/M2269 **(A)** MHOM/BR/1987/BA125 **(B)** and MHOM/BR/1989/BA336 **(C)** are shown. Images are representative of three experiments. MM - Molecular mass ladder. The original digital image of the gel is presented as Supplementary Figure S1.

The protein profiles of EVs shed by *L. amazonensis* strains showed similarities between strains MHOM/BR/1987/BA125 (Figure 3, lane B) and MHOM/BR/1989/BA336 (Figure 3, lane C) and were distinct from strain MHOM/BR/1973/M2269 (Figure 3, lane A). The EVs’ cargo contained protein fractions with molecular masses above 21 kDa, EVs derived from MHOM/BR/1987/BA125 and MHOM/BR/1989/BA336 strains exhibited prominent bands at 84, 76–66, and 62 kDa. The 76–66 kDa protein fraction showed the highest intensity. In EVs derived from the MHOM/BR/1973/M2269 strain, the most abundant fractions corresponded to 79–66 kDa, as well as 55 and 48 kDa.

**FIGURE 4:**
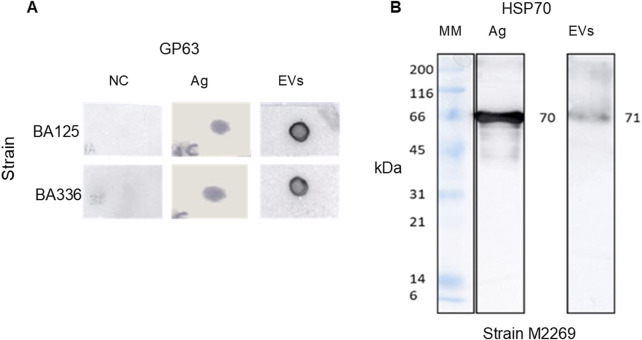
*Leishmania amazonensis-*derived EVs carry gp63 and HSP70. Digital images of nitrocellulose membrane sensitized (dot blot) with EVs or Ag of *Leishmania amazonensis* strains MHOM/BR/1987/BA125 (BA125) and MHOM/BR/1989/BA336 (BA336), or lysis buffer (NC). Membranes were incubated with anti-gp63 primary mononuclear antibody and secondary anti-mouse mononuclear antibody conjugated with peroxidase **(A)** Immunoblot of EVs from MHOM/BR/1973/M2269 strain (M2269) and Ag incubated with anti-HSP70 polyclonal antibody, followed by anti-rabbit antibody conjugated with peroxidase, is shown **(B)** Images are representative of three experiments. MM - Molecular mass ladder. The original dot blot and gel digital images are presented as Supplementary Figure S2.

Furthermore, it has been demonstrated EVs derived from cultured promastigotes of *L. amazonensis* strains contain gp63 (Figure 4A) and HSP70 (Figure 4B). Anti-HSP70 monoclonal antibody specifically recognized one EV protein fraction, indicating the presence of HSP70 in *L. amazonensis* EVs. Although both the soluble antigen and EVs exhibited bands of comparable molecular mass (70/71 kDa), the band was less intense in EVs.

Mass spectrometry analysis of *L. amazonensis-*derived EVs revealed several protein components, including contaminant proteins (e.g., keratin and trypsin) and a distinctive 26.817 kDa peptide. A search in the *UniProt_Leishmania_2022 database* of this unique peptide revealed that it matches the α-type proteasome subunit. This protein is listed in the database under the accession number A0A836KVE3 and is predicted to have the peptide sequence IF QIEYAVEAIK TPEGVVLAAEK VPSTLVIPSSMSK (Table 1). Therefore, the cargo of EVs derived from *L. amazonensis* promastigotes includes gp63, HSP70, and the α−type proteasome subunit.

**TABLE 1:** Protein identification in *Leishmania amazonensis-derived* EVs. Protein was identified by MS/MS analysis of a gel-excised band of EVs, using the *Leishmania* database. The protein accession number, number of unique peptides, and the corresponding peptide sequence are included.

Sample	Lane-03		
Database	*UniProt_Leishmania_ 2022*		
Accession number	A0A836KVE3		
Protein name	α-type proteasome subunit		
Protein score	77.22		
Coverage	15%		
Peptide	3		
Unique peptide	3		
Peptide sequence	IFQIEYAVEAIK	TPEGVVLAAEK	VPSTLVIPSSMSK
Peptide score	37.03	46.83	35.62
Unique	Y	Y	Y

### 3.3 *Leishmania amazonensis-*derived EVs are recognized by sera from experimentally infected mice and ACL patients

To examine the humoral immunogenicity of EVs emitted by *L. amazonensis,* sera (antibody titer ≥1:200) from mice experimentally infected with *L. amazonensis* presenting amastigote forms in the spleen, as well as a pool of sera samples from patients with ACL based on clinical and serological criteria, were used.

**FIGURE 5:**
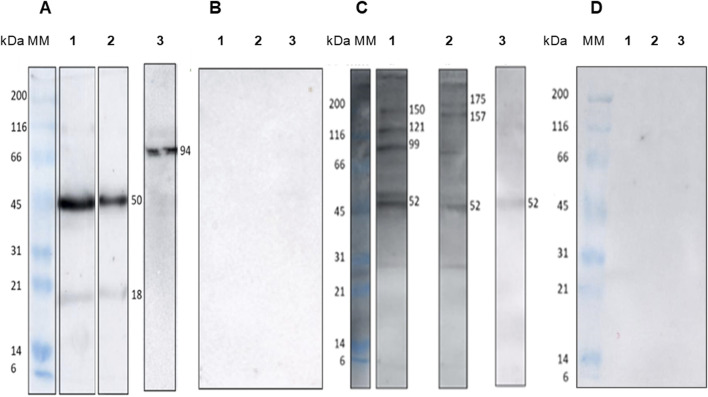
Immunogenicity of *Leishmania amazonensis-*derived EVs. Digital images of immunoblot of EVs purified from *Leishmania amazonensis* strains MHOM/BR/1987/BA125 (1), MHOM/BR/1989/BA336 (2), and MHOM/BR/1973/M2269 (3). Nitrocellulose strips were incubated with sera from mice experimentally infected with *Leishmania amazonensis* **(A)** sera from healthy mice **(B)** pooled sera of ten patients with ACL **(C)** and clinically healthy individuals **(D)**. Images are representative of three experiments. MM - Molecular mass ladder. The digital images of original immunoblots are presented as Supplementary Figure S3.

Protein fractions of 50 and 18 kDa of EVs derived from MHOM/BR/1987/BA125 and MHOM/BR/1989/BA336 strains were recognized by antibodies of *L. amazonensis-*infected BALB/c mice (Figure 5A). However, mouse sera only recognized the 94 kDa protein fraction of EVs derived from MHOM/BR/1973/M2269 strain. Serum from non-infected mice showed no reactivity towards any protein fraction (Figure 5B).

Furthermore, the protein fractions of EVs were recognized by antibodies present in the sera of patients with ACL. A 52 kDa protein fraction from EVs of all the *L. amazonensis* strains that were assessed was recognized by the sera of ACL patients. Protein fractions of 175, 150–157, 121, and 99 kDa from EVs derived from the MHOM/BR/1987/BA125 and MHOM/BR/1989/BA336 strains also reacted with human sera. By contrast, EVs from the MHOM/BR/1973/M2269 strain showed reduced reactivity with the antibodies from ACL patients (Figure 5C). Analysis of sera from healthy donors revealed no reactivity with the protein fractions of EVs (Figure 5D).

Thus, these results suggest that EVs shed by *L. amazonensis* are potentially immunogenic, as they are recognized by both experimentally infected mice and ACL-specific antibodies. However, EVs derived from MHOM/BR/1973/M2269 strain exhibit distinct antigenic characteristics.

### 3.4 EVs exert immunomodulation on host cells

To investigate the immunomodulatory effect of *L. amazonensis* EVs on immune activation of MΦs, the P388D1 murine macrophage cell line was used as a host model. This study then examined the effect of EVs shed by *L. amazonensis* promastigotes (MHOM/BR/1973/M2269 strain) on the MΦs' immune activation by analyzing the expression of genes encoding innate immune receptors and immune mediators.

**FIGURE 6:**
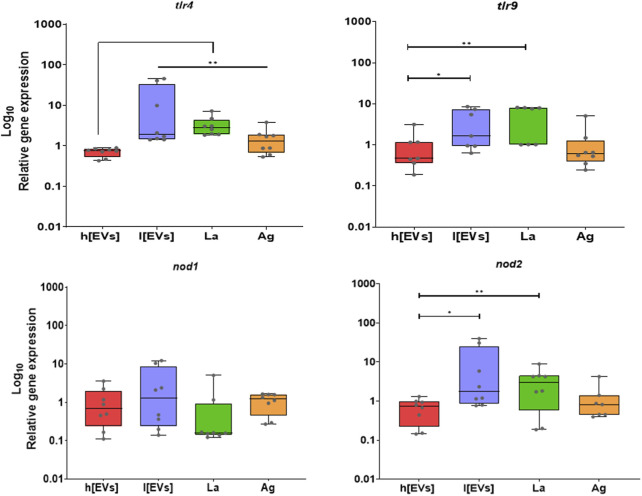
Effect of EVs on gene expression of MΦs' innate receptors. The relative gene expression of *tlr4, tlr9, nod1,* and *nod2* in MΦs stimulated with low (l [EVs], 3.54 μg.μL^-1^) or high (h [EVs], 7.09 μg.μL^-1^) concentrations of EVs derived from strain MHOM/BR/1973/M2269, or Ag, or exposed to promastigotes (LA), was determined by qPCR. Data from at least seven samples are represented by box plots showing minimum, median, and maximum values. Nonparametric Wilcoxon’s test was used for statistical comparisons, with * (*p* < 0.05) and ** (*p* < 0.01) indicating significant differences.

Lower concentrations of EVs and *L. amazonensis* parasites lead to enhanced gene expression of MΦs innate receptors (Figure 6). Compared with EVs at higher concentration, both parasites and the lower concentration of *L. amazonensis-*derived EVs induced a significant upregulation in the gene expression of *tlr4* (*p* = 0.0078), *tlr9* (*p*_l[EVs_ = 0.0156¸ *p*_La_ = 0.0078), and nod2 (p_l[EVs]_ = 0.0165, *p*_La_ = 0.0078). Additionally, parasite antigens also induced an overexpression of *tlr4* (p = 0.0078).

Therefore, parasites and lower concentrations of EVs can drive the overexpression of innate receptors on the MØ surface (TLR4), as well as intracellular innate receptors capable of binding to parasite glycosylphosphatidylinositol and CpG oligonucleotides (NOD2 and TLR9).

**FIGURE 7:**
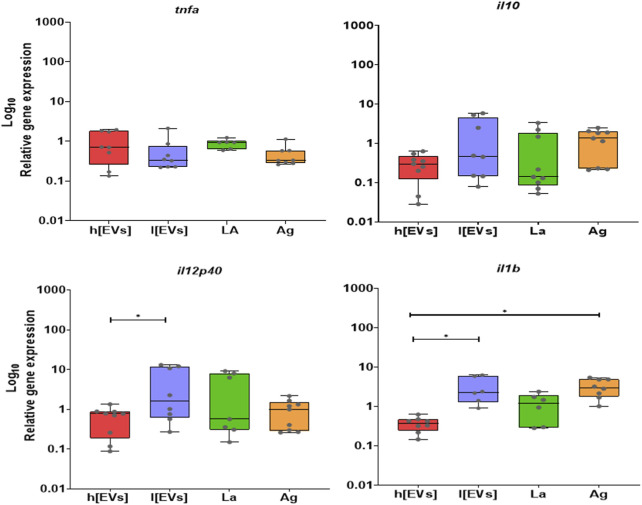
Effect of EVs on cytokine gene expression in MØs. The relative gene expression of *tnfa, il10, il12p40, and il1b* in MΦs stimulated with low (l [EVs], 3.54 μg.μL^-1^) or high (h [EVs], 7.09 μg.μL^-1^) concentrations of EVs derived from strain MHOM/BR/1973/M2269, or Ag, or exposed to promastigotes (LA), was determined by qPCR. Data from at least six samples are represented by box plots showing minimum, median, and maximum values. Nonparametric Wilcoxon’s test was used for statistical comparisons, with * (*p* < 0.05) and ** (*p* < 0.0) indicating significant differences.

Gene expression analysis of pro-and anti-inflammatory cytokines (Figure 7) revealed low *il10* and *tnf* mRNA accumulation in MΦs exposed to parasites or MΦs stimulated by parasite Ag or EVs. However, a significant upregulation of *il12p40* (*p* = 0.0391) and *il1b* (*p* = 0.0312) was found in MΦs stimulated by EVs at lower concentrations compared to MΦs induced by the higher concentration of EVs. Parasite Ag also promoted significant upregulation of *il1b* expression (*p* = 0.0078). In contrast, no significant alteration was observed in *tnfa* and *il10* expression.

Thus, these findings suggest that low concentration of EVs has the potential to induce MΦs to generate proinflammatory cytokines.

### 3.5 Macrophages maintain functionality when exposed to EVs

**FIGURE 8:**
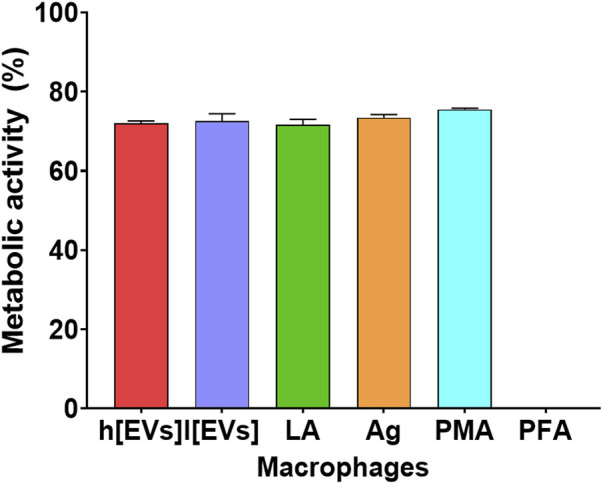
Viability of MΦs stimulated by *Leishmania amazonensis-*derived EVs. The metabolic activity of MΦs stimulated with high (h [EVs], 7.09 μg.μL^-1^) or low (l [EVs], 3.54 μg.μL^-1^) concentration of EVs derived from strain MHOM/BR/1973/M2269, or Ag, or exposed to promastigotes (LA), PMA, or paraformaldehyde (PFA, unviable cells) was determined by resazurin assay. The mean ± SEM of three samples and duplicates per sample are represented by a bar graph.

To investigate the effect of *L. amazonensis* EVs on MΦs viability, the impact on cellular metabolism was analyzed. After 24 h of incubation, there was approximately a 25% reduction in the metabolic activity (Figure 8) compared with resting cells. Regardless of the concentration used, EVs did not cause a distinct impact on the metabolic activity of MΦs, as well as live parasites and parasitic Ag, indicating that most of the cells remained viable.

Then, to evaluate the effect of EVs on the functional activity of murine MΦs, this study examined the potential of antigen presentation by analyzing the expression of surface MHC molecules and of intracellular IL-1β, which appears to be associated with *Leishmania* cutaneous lesions in mouse models. In addition, this study also assessed the balance between arginase and NO production.

**FIGURE 9:**
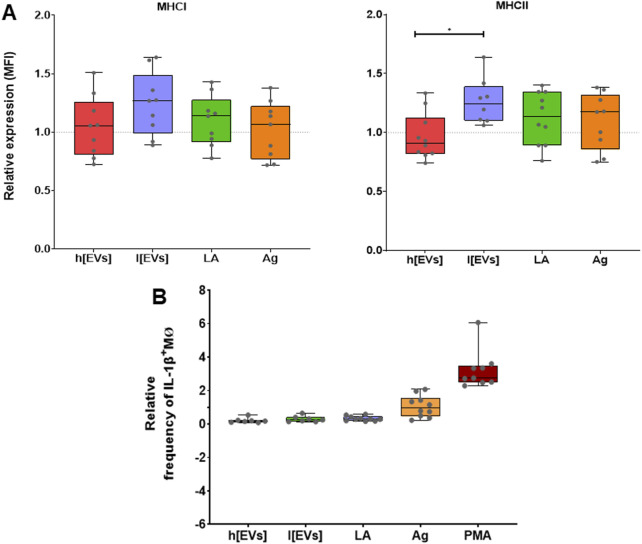
Impact of *Leishmania amazonensis-*derived EVs on MHC expression and IL-1β^+^MΦs subset*.* The mean fluorescence intensity (MFI) of MHCI and MHCII **(A)** and the relative frequency of IL-1β^+^MΦs **(B)** after stimulation with low (l [EVs], 3.54 μg.μL^-1^) or high (h [EVs], 7.09 μg.μL^-1^) concentrations of EVs derived from strain MHOM/BR/1973/M2269, or Ag, or exposed to promastigotes (LA) was determined by multiparametric flow cytometry, using monoclonal antibodies directly conjugated*.* Boxplots, including minimum, median, and maximum values, represent the results of at least seven sample normalized to resting MΦs. The dashed lines indicate the basal levels of MHC molecules or IL-1β^+^MΦs subset. Representative histograms of IL-1β^+^MΦs subset are presented as Supplementary Figure S4.

MΦs exposed to *L. amazonensis* promastigotes and MΦs stimulated by parasite antigens showed a slight increase in the expression of MHCI and MHCII molecules compared with resting MΦs. When stimulated with the lower concentration of EVs, MΦs showed the highest expression of MHCI and MHCII molecules. In contrast, MΦs stimulated with the highest concentration of EVs had the lowest levels of MHCI and MHCII molecules. Moreover, MHCII expression differed between MΦs stimulated by EVs at high versus low concentration (*p* = 0.0156) (Figure 9A). These results indicate that *L. amazonensis* EVs can modulate MΦs to express MHC molecules in a dose-dependent manner but inversely proportional.

Subsequently, the effect of parasitic EVs on IL-1β expression by MΦs was analyzed after 24 h of incubation. The proportion of IL-1β^+^MΦs subset was evaluated by multiparametric flow cytometry among MΦs stimulated by EVs at two concentrations, parasite Ag, or PMA, as well as in MΦs exposed to live promastigotes, and resting MΦs. About 1.7% of resting MΦs were IL-1β^+,^ representing a basal pro-inflammatory MΦs subset. Stimulation of MΦs with PMA, as a positive control of inflammation, led to a significant expansion in the proportion of IL-1β^+^MΦs (*p* = 0.0019), demonstrating that cells were functional and able to produce this pro-inflammatory immune mediator (Figure 9B). MΦs stimulated with parasite Ag showed a frequency of IL-1β^+^MΦs (1.66%) similar to resting MΦs, indicating that the soluble antigens did not alter the MΦs phenotype. MΦs stimulated with EVs at 7 and 3.5 μg.μL^-1^ presented 0.45% (*p* = 0.0078) and 0.52% (*p* = 0.0016) of IL-1β^+^MΦs cells, respectively, which were significantly lower when compared with the resting MØs. Similarly, only 0.55% of MΦs exposed to live promastigotes were positive for IL-1β, which differs significantly from resting cells (*p* = 0.0009). These data indicate that *L. amazonensis*-derived EVs and promastigotes prevent murine MΦs from expressing IL-1β.

**FIGURE 10:**
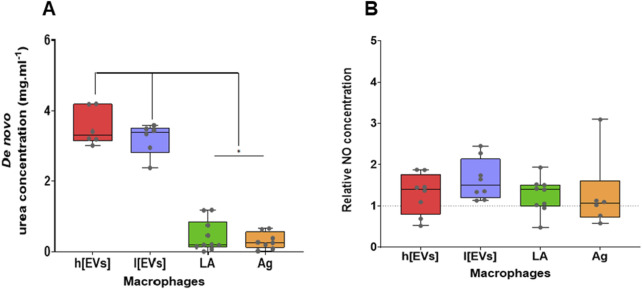
Effect of *Leishmania amazonensis* EVs on the MΦs' production of urea and NO. *De novo* urea production **(A)** and NO **(B)** released by MΦs stimulated by low (l [EVs], 3.54 μg.μL^-1^) or high (h [EVs], 7.09 μg.μL^-1^) concentrations of EVs derived from strain MHOM/BR/1973/M2269, or Ag or exposed to promastigotes (LA), were estimated using colorimetric assays. NO values were normalized to resting MΦs. Data from at least six samples are represented as box plots, including minimum, median, and maximum values. The dashed line represents basal NO levels.

The levels of urea and NO produced by MΦs stimulated by *L. amazonensis-*derived EVs or parasite Ag and exposed to parasites were quantified after 24 h of incubation. MΦs exposed to *L. amazonensis* promastigotes and Ag-stimulated MΦs produced the lowest urea levels (Figure 10A). In contrast, higher levels of urea *de novo* produced were found in MΦs stimulated by EVs (*p* = 0.031). Regarding NO release, MΦs stimulated by the EVs and parasite antigens or exposed to parasites produced NO levels comparable to the basal levels exhibited by resting MΦs (Figure 10B), Therefore, these findings suggest that EVs activate arginase in MØs, leading to the hydrolysis of arginine to produce urea without affecting steady-state NO production.

## 4 Discussion

Extracellular vesicles are naturally released by eukaryotic cells into the surrounding microenvironment and are recognized as part of a system of cell-to-cell communication that interferes with the activity of recipient cells. Operating as macromolecule carriers, EVs play a crucial role in various cellular processes, including inflammation, cell proliferation, and the immune response. Moreover, EVs are being explored for the use in the development of diagnostic tools, as well as for therapeutic and prophylactic applications for several diseases (Montaner-Tarbes et al., 2021; Chowdhury et al., 2024; Peregrino et al., 2024; Zhang et al., 2024; Santos and Almeida, 2021). The release of EVs by parasites adds a new dimension to cell-to-cell communication, by facilitating both inter (parasite-to-parasite) and intraspecific (parasite-to-host) interactions (Marcilla et al., 2014). Therefore, the current study investigated the immunopathogenic effect of EVs derived from *L. amazonensis* promastigotes by examining their protein profile, evaluating their immunogenicity, and assessing their impact on MΦs activity.

The MISEV 2023 guidelines from the International Society of Extracellular Vesicles (ISEV) emphasize the importance of standardized EVs’ preparation, including the composition of culture media, as complex supplements like blood serum are rich in EVs. When culture supplements containing EVs cannot be avoided, the use of EVs-depleted supplements is recommended (Welsh et al., 2024). As the non-supplementation of *L. amazonensis* axenic culture medium with FBS greatly reduces parasite viability and impairs the quality of EV purification, using exofree FBS (EVs-depleted FBS) was the best alternative to obtain parasite EVs. To validate EVs extraction, a combination of detection methods is recommended, including analysis of shape, size, and concentration. NTA and protein measures are the most widely used quantitative methods in addition to EV images (Welsh et al., 2024). The TEM images revealed the classic characteristics of *L. amazonensis* EVs (Jung and Mun, 2018), and the size measurements aligned with the NTA results. Therefore, two types of nanoscale EVs were identified: small vesicles (<100 nm), which are compatible with exosomes that have an endosomal origin, and larger vesicles (>100 nm), which are identified as microvesicles that are shed directly into the extracellular space from the promastigote. Similar findings have recently been described for other cutaneous *Leishmania* species (Fernandez-Becerra et al., 2023).

The molecular composition of parasite-derived EVs has been shown to contain molecules that can increase the success of infection. More than 300 molecules have been identified in EVs shed by *Leishmania* parasites, including virulence factors (e.g., gp63, HSP10, HSP70, TRYP1, 14-3-3-like protein, STI1 stress-induced protein), as well as LPG, and elongation factor 1 (EF-1) (Silverman et al., 2010; Hassani et al., 2011; Olivier et al., 2012; Hosseini et al., 2013; Barbosa et al., 2018). The current study reveals that EVs’ protein profile exhibits a wide range of protein fractions and some variations among the analyzed *L. amazonensis* strains. Strains MHOM/BR/1987/BA125 and MHOM/BR/1989/BA336 exhibit considerable similarity, whereas strain MHOM/BR/1973/M2269 shows several differences. These variations detected in SDS-PAGE profile, as well as in immunoblot using serum from infected mice and humans, may be attributed to isomeric forms, the presence of more than one protein in each fraction, or variations in protein concentration, which could be related to the time the parasites were maintained *in vitro*.

*Leishmania amazonensis* EVs carry key proteins like HSP70 and the zinc metalloprotease gp63 (63–68 kDa). HSP70 is a molecular chaperone that protects other molecules from degradation when facing stress conditions. In *Leishmania* parasites, this cytoplasmic protein confers protection against heat shock during the transition from sandfly vector to the mammalian host and also when dealing with oxidative stress, thereby preserving protein homeostasis. *Leishmania* parasites, mainly the promastigote form, have a surface that is densely covered by gp63 (Etges et al., 1986; Bouvier et al., 1987; Chang et al., 1990; Hey et al., 1994; Pandey et al., 2004; Woelbing et al., 2006), which plays a crucial role in parasite survival by inactivating complement factors. It can convert the complement factor 3b (C3b) into an inactive form (C3bi), thereby preventing complement-mediated lysis and facilitating parasite phagocytosis, enabling parasite survival within the MΦs (Liese et al., 2008). In *L. mexicana* promastigotes, gp63 favors the degradation of extracellular matrix proteins via MФs signaling mechanisms, further promoting parasite survival (McGwire and Satoskar, 2014). Moreover, gp63 has been reported to confer protection to the amastigote form in the harsh phagolysosome environment of MΦs (Chen et al., 2000). Through its proteolytic properties, gp63 interferes with host signaling pathways, altering their related functions (Isnard et al., 2012), including the cleavage and degradation of kinases and transcription factors. This leads to the inhibition of relevant enzymatic activities (Olivier et al., 2012; Chaudhuri et al., 1988; Chaudhuri et al., 1989; Santos et al., 2006; Lieke et al., 2008; Moradin and Descoteaux, 2012). In a previous study, Silva Lira Filho and colleagues (Silva Lira Filho et al., 2022) reported that *L. amazonensis* EVs carrying gp63 contributed to the cutaneous inflammatory response against infection. Therefore, the detection of both gp63 and HSP70 in EVs isolated from parasites grown under conditions that minimize contamination by other EVs, strongly supports that EVs originate from *L. amazonensis* promastigotes. Moreover, *L. shawi* and *L. guyanensis*-derived EVs also appear to include other active proteases in their cargo (Weber et al., 2023).

The findings of the current study indicate that α-type proteasome subunit also makes part of the cargo of *L. amazonensis*-derived EVs. The proteasome is a multi-catalytic protease complex characterized by its ability to cleave peptides. It is present in the cytoplasm of eukaryotic cells (Djaballah et al., 1993) and in protozoa like *Leishmania* (Silva-Jardim et al., 2004). This large structure consists of a 20S proteolytic core, which is capped on one or both ends by a 19S regulatory particle (Antoine et al., 1999; Schmidtke et al., 2000). The proteasome plays a crucial role in protein degradation, particularly during stress responses. The α-type proteasome subunit has been identified previously in *L. martiniquensis* (Almutairi et al., 2021) and appears to be essential for parasite survival and growth (Moradin and Descoteaux, 2012). Thus, the delivery of the α-type proteasome subunit by EVs to host recipient cells may introduce changes in cellular activities that promote parasite survival. Taken together, these findings suggest that *L. amazonensis-*derived EVs may interact with host cells, contributing to the protection of intracellular parasites and modulating host cell activity, favoring the successful establishment of infection.

The antigenic composition of *L. amazonensis-*derived EVs was recognized by antibodies from experimentally infected mice and naturally infected humans, suggesting that EVs are immunogenic and can elicit a humoral immune response. Furthermore, exposure of murine MФs to EVs did not significantly affect their viability but did induce the expression of innate immune receptor genes and the expression of MHC molecules. At lower concentrations, EVs promote the gene expression of plasma membrane TLR4 and endosomal TLR9. Signaling these receptor pathways can drive the upregulation of pro-inflammatory cytokine genes, which are essential for promoting Th1-mediated immunity.

Indeed, in the present study, the upregulation of *tlr4* and *tlr9* was associated with the increased levels of *il12p40*, which encodes a subunit of IL-12 that is a key cytokine involved in Th1 immune response (Sharma et al., 2024). However, Sauter and colleagues (Sauter et al., 2019) reported that TLR9 signaling mediated by DNA of amastigotes *and* microvesicles of *L*. *amazonensis* inhibits NO production. Consistently, in the current study, MФs exposed to *L. amazonensis*-derived EVs or exposed to *L. amazonensis* parasites also failed to release NO. These findings highlight that *EVs can modulate the* expression of innate receptors, thereby inhibiting MФs' microbicidal activity, which has implications for disease severity. Moreover, NOD1 and NOD2 signaling may influence the production of pro-inflammatory cytokines. While activation of the NOD1 pathway can promote the production of TNF-α, NOD2 can lead to the synthesis of IL-1β, which can favor the inflammasome activation.

A previous report (dos Santos et al., 2017) indicates that *L. amazonensis* mRNA induces the upregulation of *NOD2* but not *NOD1,* which is in line with the findings of the present study*.* The upregulation of *NOD2* by lower concentrations of EVs is associated with an increase in *il1β* gene expression, which plays a key role in driving inflammatory response essential for defending against infections. While it is well established that different species of *Leishmania* elicit diverse host immune responses, exposure of human MΦs to *L. major* parasites has been shown to increase IL-1β production (Patil et al., 2018). In contrast, dendritic cells infected with *L. amazonensis* exhibit an anti-inflammatory phenotype, characterized by reduced IL-1β production, which is influenced by epigenetic and transcriptional regulation involving the nuclear factor kappa B (Lecoeur et al., 2020).

On the other hand, Weber and colleagues (Weber et al., 2023) reported that the upregulation of *il1b* in murine MΦs stimulated by EVs derived from *L. shawi* and *L. guyanensis* is influenced by both the duration of stimulation and the concentration of EVs. In the current study, although *L. amazonensis-*derived EVs, promastigotes, and parasite Ag favor the upregulation of *il1b*, the contraction of IL-1β^+^MΦs subset reflects a reduced number of macrophages expressing IL-1β, pointing to a possible regulation of IL-1β translation. The apparent discrepancy between the high levels of *il1b* mRNA observed in MΦs stimulated with EVs and Ag, and the low levels of MΦs expressing pro-IL-1β, may indicate translational repression (potentially involving the intervention of microRNAs), or the rapid processing of pro-IL-1β into its active form. In contrast, the positive control (MΦs stimulated with PMA) exhibited both high *il1b* mRNA levels (mean ≈5) and accumulation of pro-IL-1β. Taken together, these findings support the possibility that EVs and Ag interfere with the translation of IL-1β. As this cytokine is one of the earliest cytokines produced during infection and plays a crucial role in inducing the release of IL-12, its low availability might impair the early activation of an optimal cellular immune response.

MΦs are APCs that activate the adaptive immune response by presenting *Leishmania* antigens to CD4^+^ T lymphocytes through MHCII molecules. Furthermore, MФs can also cross-present antigens to CD8^+^ T lymphocytes through MHCI molecules. Interference in antigen presentation mechanisms, by inhibiting MHC expression, is one of the parasite’s methods to avoid the detrimental effects of the host immune response (Matheoud et al., 2013). It has already been demonstrated that MHCII molecules of *L. amazonensis* infected MØs stimulated by IFN-γ were retained in the parasitophorous vacuole. The internalization and degradation of these molecules impair antigenic presentation to T cells. Therefore, the concentration of MHCII molecules in the phagosome is recognized as a survival strategy employed by *Leishmania* parasites to subvert antigenic presentation mechanisms and impair the activation of the adaptive immune response (Antoine et al., 1999). In the current study, a low concentration of *L. amazonensis*-derived EVs promoted the overexpression of MHCII at MФ surface, favoring antigenic presentation to CD4^+^ T cells. In contrast, *L. donovani* exosomes were shown to inhibit MHCII expression in human monocyte-derived dendritic cells (Marcilla et al., 2014). On the other hand, more recent studies have shown that murine MΦs stimulated by EVs shed by *L. infantum*, *L. shawi,* and *L. guyanensis* parasites increased the expression of MHCI molecules and that *L. amazonensis* and *L. infantum-*derived EVs lead to overexpression of MHCI by canine monocyte-derived dendritic cells (Weber et al., 2023; Pérez-Cabezas et al., 2018; Valério-Bolas et al., 2024). Interestingly, in the current study, higher concentrations of *L. amazonensis-*derived EVs appear to impair gene expression of innate receptors and cytokines, as well as MHC expression in murine MΦs. The interaction of *L. mexicana* exosomes and murine MØs led to the inhibition of MHCI expression (Soto-Serna et al., 2020). This inhibition may be linked to gp63, which impairs the presentation of *Leishmania* antigens to CD8^+^ T cells through MHCI molecules (Patil et al., 2018).

During the early stage of MΦs infection, *Leishmania* gp63 inactivates the vesicle-associated membrane protein 8 (VAMP8), which is a key protein of MΦs membrane involved in vesicle trafficking. This disruption alters the pH of the phagosome, reducing proteolytic activity and antigenic processing that leads to lower MHCI expression, resulting in impaired activation of cytotoxic T cells (Matheoud et al., 2013). These cells play a crucial role in eliminating infected cells. Thus, the downregulation of antigen-presenting mechanisms by parasite EVs can limit the functional MΦs' capacity to activate the adaptive immune response and can represent an additional survival strategy of *Leishmania*.

Taken together, these findings highlight that lower concentrations of EVs promote the controlled immune activation of murine MΦs by triggering innate receptor signaling and generating IL-12 along with the overexpression of MHCII molecules, which suggests antigen presentation to lymphocytes. In contrast, higher concentrations of EVs lead to immune suppression by downregulating innate receptors and immune mediators, thereby impairing antigen presentation, probably by delivering high amounts of suppressive parasite antigens. These findings are consistent with a biphasic immune response, in which low doses stimulate, and high doses suppress cellular functions.

Pro-inflammatory MФs constitute the first innate line of defense against intracellular pathogens. These MФs are activated by the presence of IFN-γ and TNF-α, or *Leishmania* lipophosphoglycan (LPG) (Ibraim et al., 2013), leading to the production of pro-inflammatory cytokines and reactive oxygen species (Atri et al., 2018), which drive an inflammatory response that is crucial for host-defense against infection. Inflammatory MΦs can eliminate intracellular parasites by producing NO, which is an antimicrobial agent that directly destroys parasites and prevents tissue damage by restraining the recruitment of monocytes/macrophages, which are the definitive *Leishmania* host cells (Formaglio et al., 2021). On the other hand, pre-resolving inflammation and tissue repair MΦs produce urea and ornithine, which are involved in the biosynthesis of polyamines that are beneficial for parasite growth. These metabolites are also essential for collagen synthesis and cell proliferation, contributing to tissue repair and healing (Naderer and McConville, 2008).

Studies conducted with *L. braziliensis* and *L. amazonensis* identified a predominance of anti-inflammatory MΦs associated with chronic disease (Sandoval P et al., 2021; Silva et al., 2019). Thus, in the current study, EVs were found to induce murine MΦs to produce urea while maintaining low NO levels, which are associated with the restraining of IL-1β^+^MΦs subset and potential generation of IL-12. Thus, *L. amazonensis-*derived EVs direct rodent MΦs toward a non-polarized state. Taken together, these findings suggest that EVs from *L. amazonensis* impair MΦs polarization, favoring urea production that is favorable to parasite growth associated with the transcription of pro-inflammatory immune mediators that may limit parasite survival, supporting the establishment of a chronic infection.

While the current study provides valuable insights, the limitations of how EVs modulate host cells remain unclear. Further *in vitro* research is needed to deepen the understanding of the effects of parasitic EVs. Moreover, *in vivo* validation of EVs’ immune modulation is an essential and relatively unexplored area of research that is key to fully elucidating the role of EVs in the host’s immune response.

In conclusion, EVs shed by *L. amazonensis* promastigotes carry proteins that can vary across parasite strains. Furthermore, depending on the amount, EVs can influence the immunopathology of cutaneous leishmaniasis by modulating the immune activity of MΦs, which are both the first line of host innate immune response and the *Leishmania* definitive host cell. Modulation of MΦs' activity can affect the development of an effective adaptive immune response. In addition, EVs can induce arginase activity, leading to the hydrolysis of arginine into urea and ornithine, which are involved in tissue regeneration and support skin homeostasis. This process is important for controlling the immunopathology of ACL caused by *L. amazonensis* and contributes to the establishment of a chronic infection. Although future research is needed to fully understand the effect of EVs on the various aspects of MΦs' activity, the immunomodulatory characteristics of *L. amazonensis-*derived EVs appear to have the potential to advance the implementation of new strategies to control ACL. These include the development of EV-based vaccines and the design of innovative immune therapeutic approaches targeting host-parasite interactions.

## Data Availability

The raw data supporting the conclusions of this article will be made available by the authors, without undue reservation.
