# Supplementary figures and images for: Unleashing the immune modulatory potential of Leishmania amazonensis-derived extracellular vesicles in American cutaneous leishmaniasis
Source: Front Mol Biosci. 2025 Sep 22;12:1593363. doi: 10.3389/fmolb.2025.1593363 (PMC12497982; doi:10.3389/fmolb.2025.1593363)

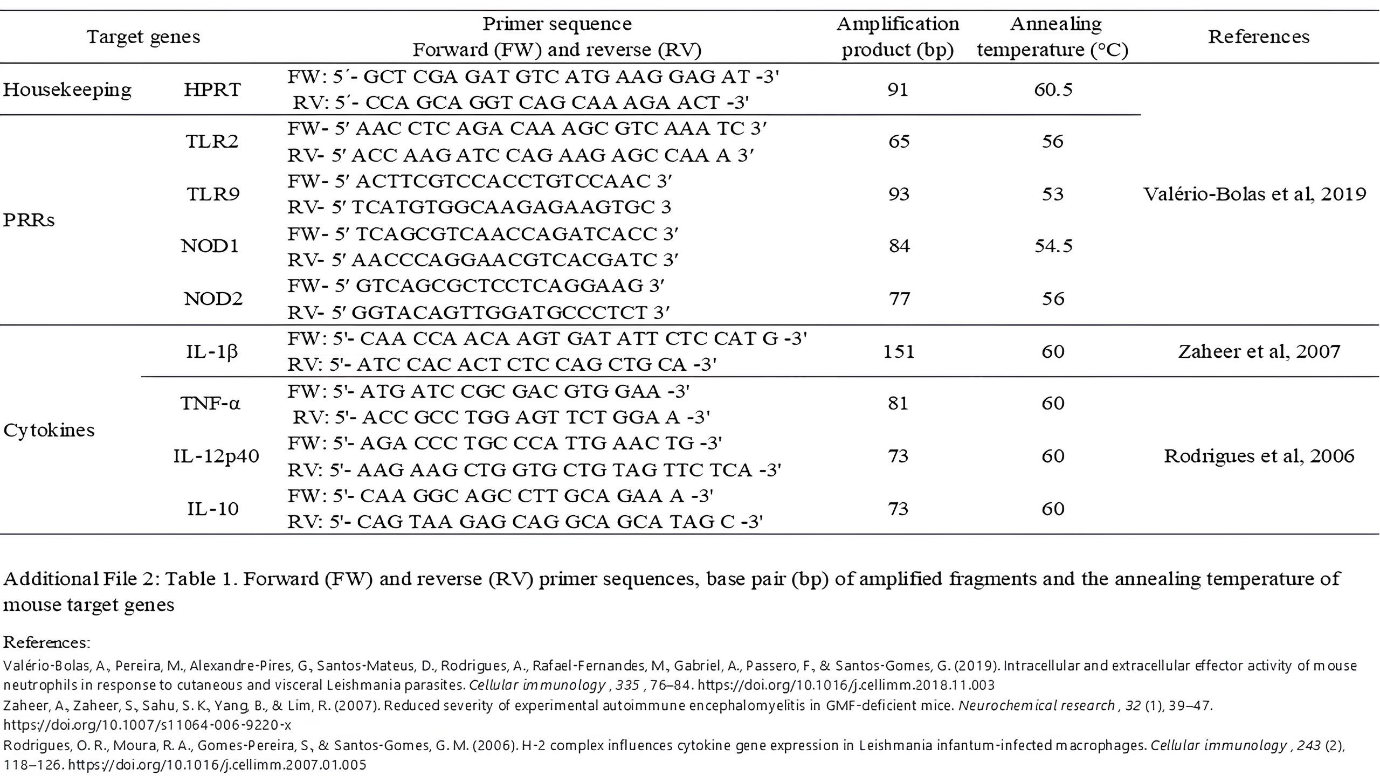

Supplement: Supplementary file 1 [file Table1.docx]

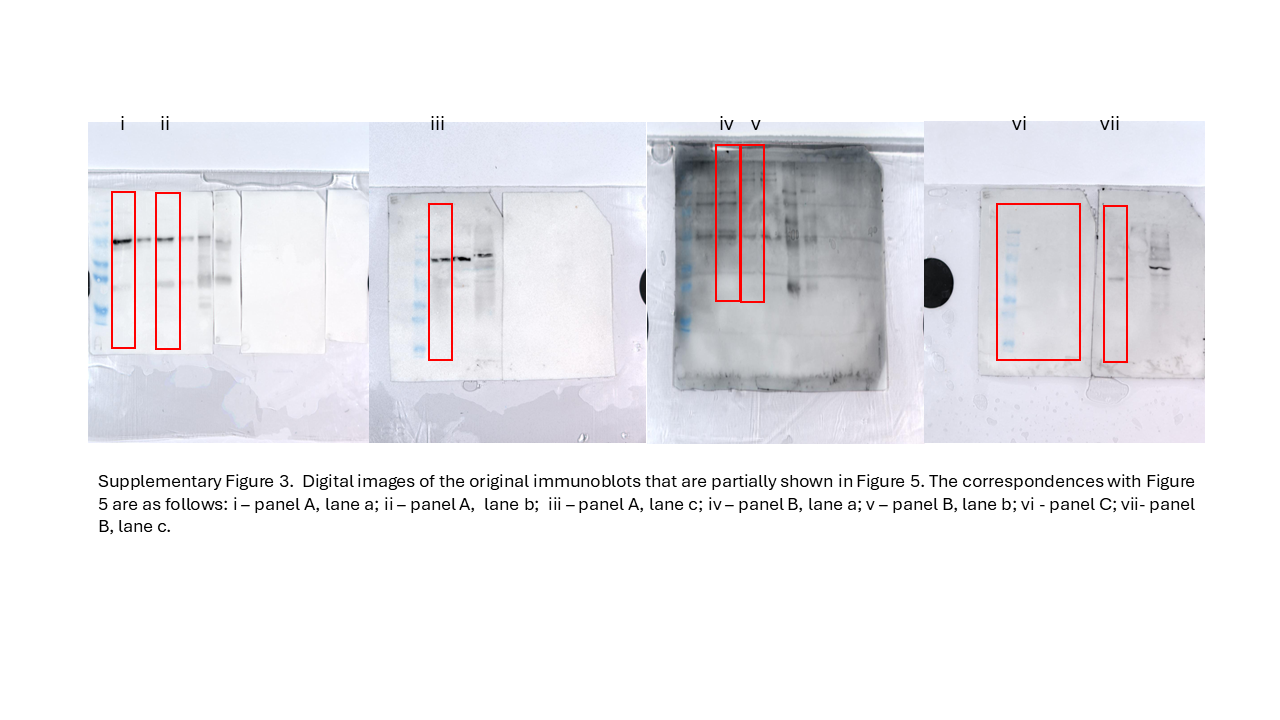

Supplement: Supplementary file 2 [file Image3.tif]

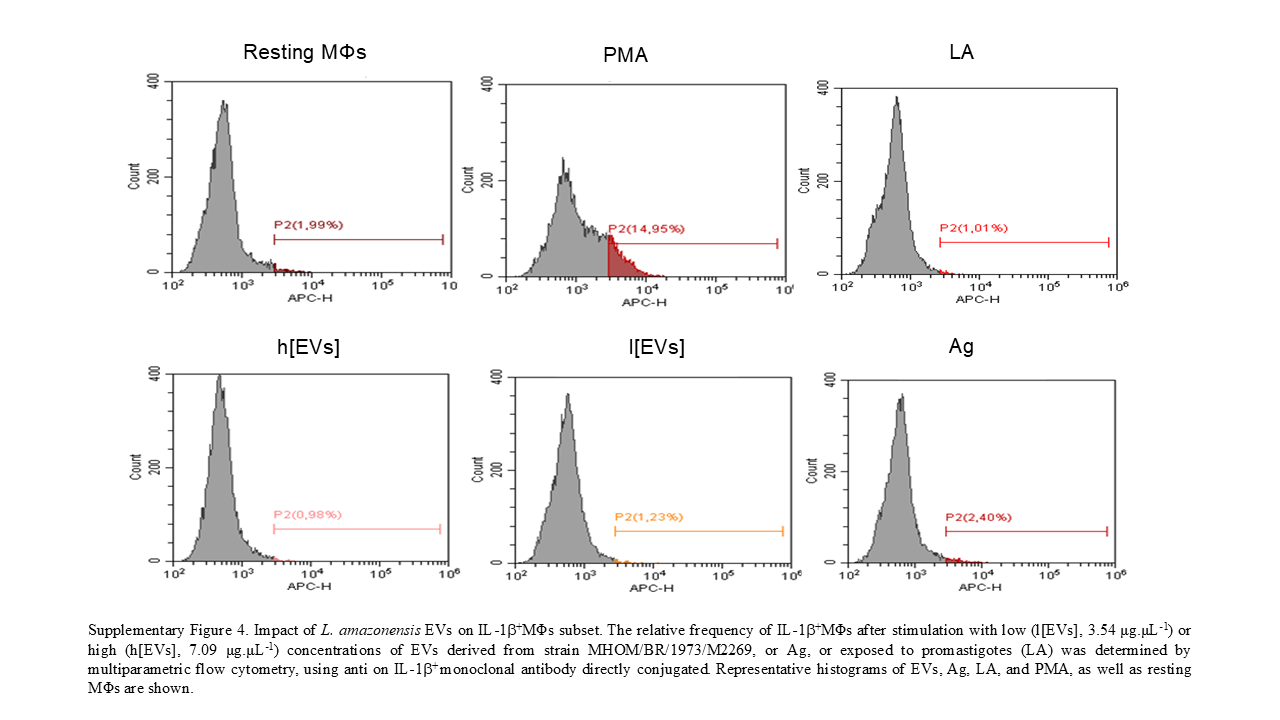

Supplement: Supplementary file 3 [file Image4.tif]

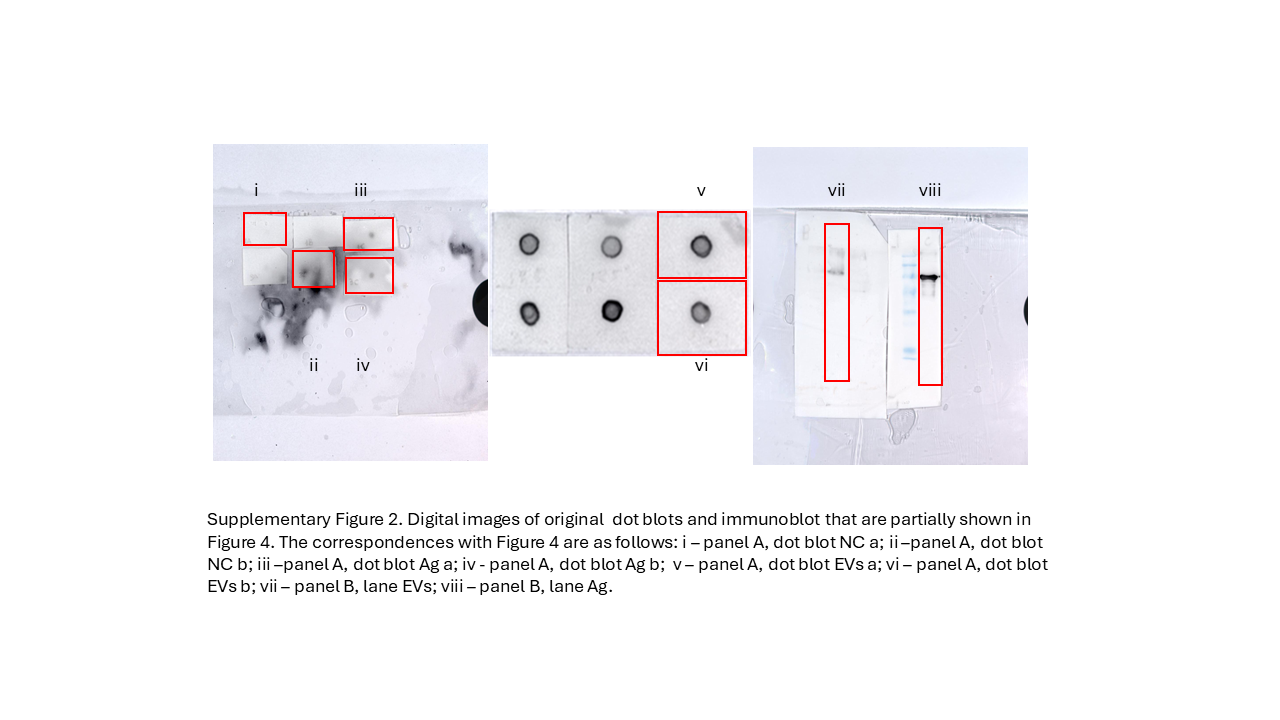

Supplement: Supplementary file 4 [file Image2.tif]

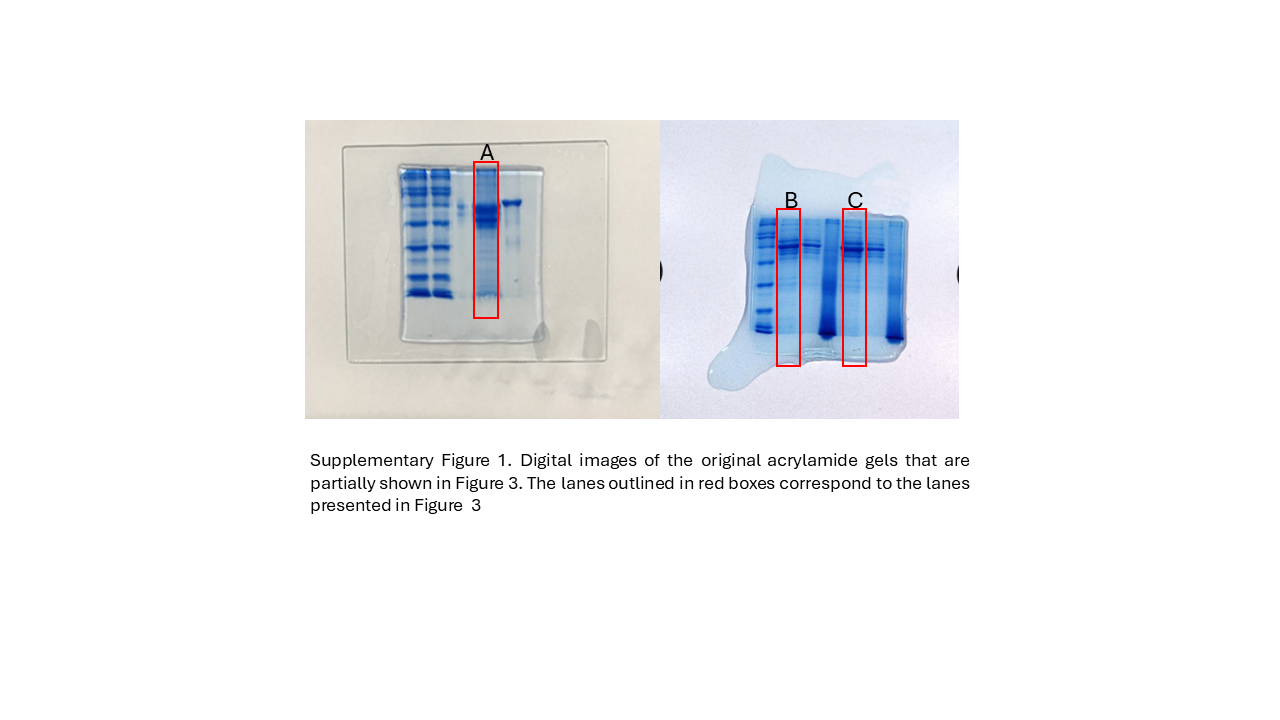

Supplement: Supplementary file 5 [file Image1.tif]

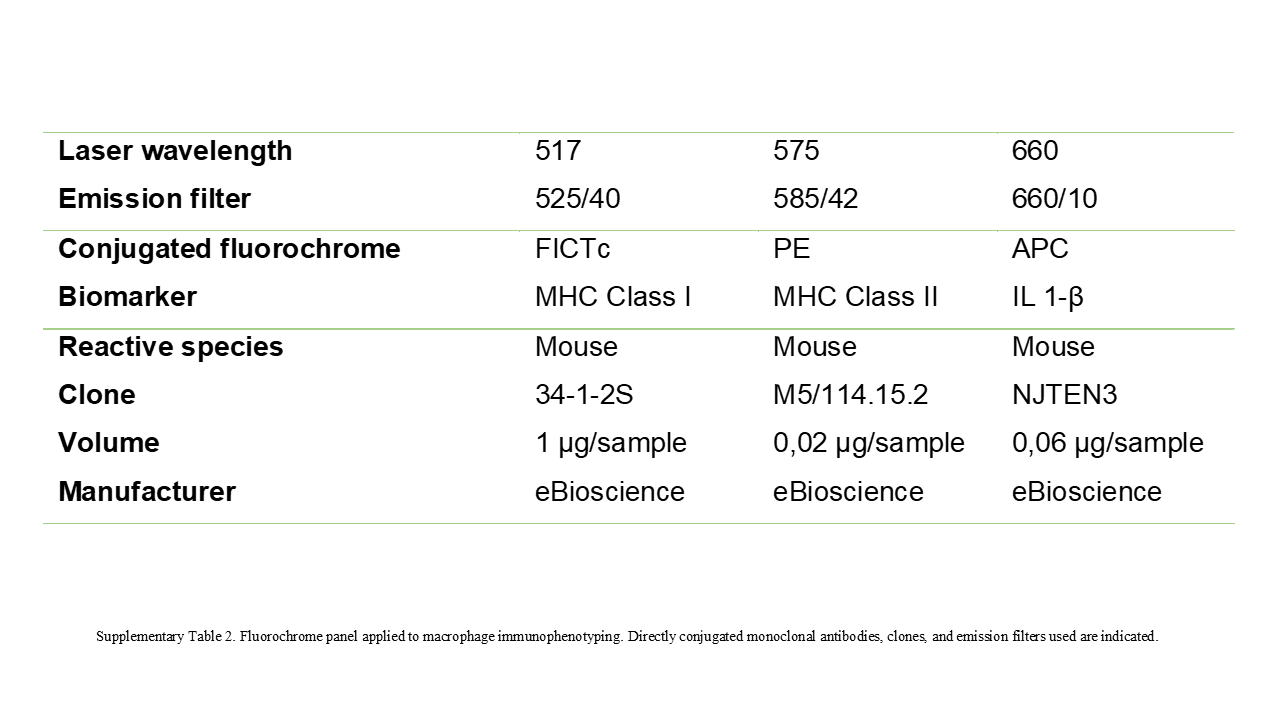

Supplement: Supplementary file 6 [file Table2.docx]
